# The Fractal Geometry of the Nymphalid Groundplan: Self-Similar Configuration of Color Pattern Symmetry Systems in Butterfly Wings

**DOI:** 10.3390/insects12010039

**Published:** 2021-01-06

**Authors:** Joji M. Otaki

**Affiliations:** The BCPH Unit of Molecular Physiology, Department of Chemistry, Biology and Marine Science, Faculty of Science, University of the Ryukyus, Okinawa 903-0213, Japan; otaki@sci.u-ryukyu.ac.jp; Tel.: +81-98-895-8557

**Keywords:** butterfly wing, color pattern, color pattern element, fractal, Lepidoptera, nymphalid groundplan, Nymphalidae, organizer, self-similarity, serial induction, symmetry system, symmetry breaking

## Abstract

**Simple Summary:**

Highly diverse color patterns of butterfly wings can be explained as modifications of an archetypical color pattern of nymphalid butterflies called the nymphalid groundplan. The nymphalid groundplan contains three major symmetry systems and a discal symmetry system, but their relationships have been elusive. Here, the morphological and spatial relationships among these symmetry systems were studied based on cross-species color-pattern comparisons of the hindwings in nymphalid butterflies. It was shown that all symmetry systems can be expressed as various structures, suggesting the equivalence (homology) of these systems in developmental potential. In some cases, the discal symmetry system is circularly surrounded by the central symmetry system, which may then be surrounded by the border and basal symmetry systems, indicating a unified supersymmetry system covering the entire wing. These results suggest that butterfly color patterns are hierarchically constructed; one system is nested within another system, which is a self-similar relationship that achieves the fractal geometry. This self-similarity is likely mediated by the serial induction of organizers during development, and a possible mechanism is proposed for symmetry breaking of the system morphology, which contributes to the diversity of butterfly wing color patterns.

**Abstract:**

The nymphalid groundplan is an archetypical color pattern of nymphalid butterflies involving three major symmetry systems and a discal symmetry system, which share the basic morphogenesis unit. Here, the morphological and spatial relationships among these symmetry systems were studied based on cross-species comparisons of nymphalid hindwings. Based on findings in *Neope* and *Symbrenthia*, all three major symmetry systems can be expressed as bands, spots, or eyespot-like structures, suggesting equivalence (homology) of these systems in developmental potential. The discal symmetry system can also be expressed as various structures. The discal symmetry system is circularly surrounded by the central symmetry system, which may then be surrounded by the border and basal symmetry systems, based mainly on findings in *Agrias*, indicating a unified supersymmetry system covering the entire wing. The border symmetry system can occupy the central part of the wing when the central symmetry system is compromised, as seen in *Callicore*. These results suggest that butterfly color patterns are hierarchically constructed in a self-similar fashion, as the fractal geometry of the nymphalid groundplan. This self-similarity is likely mediated by the serial induction of organizers, and symmetry breaking of the system morphology may be generated by the collision of opposing signals during development.

## 1. Introduction

The history of biological sciences is replete with the tactful use of model organisms with particular unique characters. One such well-established model organism is the fruit fly *Drosophila melanogaster*. This insect has contributed to genetics and developmental biology since the early 20th century [1]. There has been intensive use of butterflies in developmental biology since the late 20th century [2], but much interest was placed on comparative analyses of butterfly wing color patterns in the early 20th century, as illustrated by the independent proposition of the nymphalid groundplan (NGP) by Schwanwitsch (1924) [3] and Süffert (1927) [4]. Although few historical records have been found, Fritz Süffert was a member of the laboratory of Hans Spemann [5], who established key concepts in developmental biology, such as the organizer, induction, and morphogen [6,7]. In this line of developmental studies, Süffert (and Schwanwitsch) likely aimed to understand the developmental mechanisms of color pattern determination in butterflies. The modern version of the nymphalid groundplan was proposed by Nijhout [2,8], and minor points of the groundplan were later revised by Otaki [9,10,11,12].

**Figure 1:**
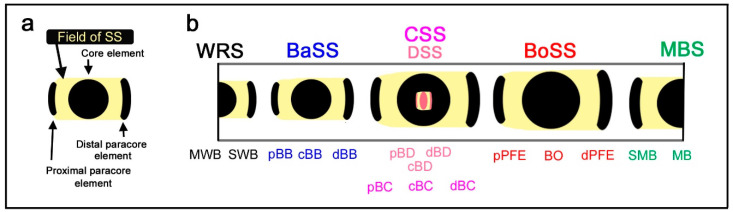
Simplified schematic representation of the nymphalid groundplan (NGP). For the most recent wing shape version of the nymphalid groundplan, see Figure 1 in the previous study [12]. (**a**) General system configuration. This is the basic morphogenesis unit of the nymphalid groundplan. A core element is located at the center, and a pair of paracore elements are located on the distal and proximal sides of the core element. The area between the distal and proximal paracore elements is considered the field of the symmetry system. (**b**) The entire nymphalid groundplan. The distal side is to the right, and the proximal side is to the left. The full basic morphogenesis unit is repeated three times to construct the basal, central, and border symmetry systems, although the status of the basal symmetry system is tentative. In addition, the discal symmetry system is located within the central symmetry system. The half basic morphogenesis unit is repeated twice to construct the marginal band system and the wing root band system. Abbreviations: WRS: wing root band system, BaSS: basal symmetry system, CSS; central symmetry system, DSS: discal symmetry system, BoSS: border symmetry system, MBS: marginal band system, MWB: marginal wing root band, SWB: submarginal wing root band, pBB: proximal band of the basal symmetry system, cBB: central band of the basal symmetry system, dBB: distal band of the basal symmetry system, pBC: proximal band of the central symmetry system, cBC: central band of the central symmetry system, dBC: distal band of the central symmetry system, pBD: proximal band of the discal symmetry system, cBD: central band of the discal symmetry system, dBD: distal band of the discal symmetry system, pPFE: proximal parafocal element, BO: border ocellus, dPFE: distal parafocal element, SMB: submarginal band, MB: marginal band.

The essence of the nymphalid groundplan can be reduced to a set of color pattern elements, including a core element at the center and a pair of paracore elements on the distal and proximal sides of the wing (Figure 1a) [10]. This set of elements may be considered the basic morphogenesis unit [10] because it is likely repeated a number of times to construct the entire wing pattern. In other words, a symmetry system is constituted by a modular set of the basic morphogenesis unit. The concept of the morphogenesis unit is a generalization of the relationship between an eyespot and parafocal elements [13,14,15,16,17,18], and the nymphalid groundplan is likely composed of repetitions of this unit. However, this aspect of the nymphalid groundplan has not been examined sufficiently in the literature.

**Figure 2:**
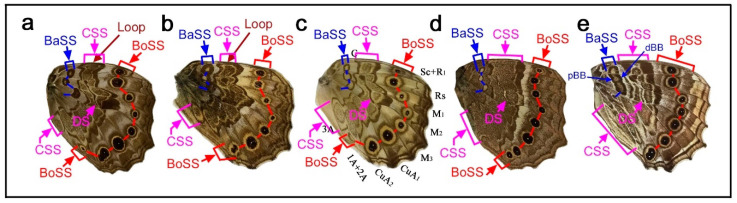
The central and basal symmetry systems (CSS and BaSS) in comparison to the border symmetry system (BoSS) in the genus *Neope*. Spots of the basal symmetry system are connected with blue lines, and the eyespots (border ocelli) of the border symmetry system are connected with red lines. DS indicates the discal spot. (**a**) *Neope bremeri*. Anterior connection of the distal and proximal bands of the central symmetry system is indicated as the Loop (also in b). (**b**) *Neope niphonica*. (**c**) *Neope goschkevitschii*. The names of the wing compartments are indicated. (**d**) *Neope muirheadi*. (**e**) *Neope yama*. The basal symmetry system (BaSS) has the distal and proximal bands (dBB and pBB).

In the nymphalid ground plan, there are three major symmetry systems: the central symmetry system (CSS) at the center, the border symmetry system (BoSS) on the distal side, and the basal symmetry system (BaSS) on the proximal side (Figure 2b). Symmetry systems are so named because its color pattern is symmetric (color symmetry rule), but its morphological structure is not [2]. This asymmetry can be considered as one of the bases of color pattern diversity in butterflies. The major symmetry systems often transverse the entire wing surface from the anterior (costal) to posterior (hind) margins as bands or serial spots with a possible exception of the basal symmetry system. In the nymphalid groundplan proposed in previous studies [2,3,4,8,9,10,12], the border symmetry system includes serial eyespots referred to as border ocelli (BO), and other symmetry systems do not, which (probably misleadingly) suggests that the three systems are qualitatively different and completely unrelated. The basal symmetry system is especially small and may be depicted as a short narrow band in the nymphalid groundplan. The most recent revision of the nymphalid groundplan established the discal symmetry system as an additional symmetry system that is nested within the central symmetry system [12]. 

An unsolved problem in the nymphalid groundplan is the status of the second discal spot (DII) often located just proximal to the first discal spot (DI; the discal symmetry system). Schwanwitsch (1924) [3] identified DII as the equivalent system for the basal symmetry system identified by Süffert (1927) [4] and Nijhout (1991) [2]. DII has been proposed to be a serial homolog of DI, based on molecular and comparative studies; the expression of DII is correlated with that of DI [19,20,21]. DII may be a special case of the basal symmetry system, but alternatively and more likely, DII may be different from the basal symmetry system, as the discal spot is nested within but different from the central symmetry system [12].

In addition to these symmetry systems, there are two peripheral systems: the marginal band system (MBS) along the outer (distal) wing margin and the wing root band system (WRS) near the wing base. The marginal band system has been considered a half-symmetry system, in contrast to the full symmetry of the major systems, because organizers of the peripheral systems are located at the wing edge [11]. Similarly, it can be speculated that the wing root band system may be a half-symmetry system [9,10,11,12].

What kinds of relationships may be found among these symmetry systems? Some nymphalid butterflies, including those of the genera *Charaxes* and *Heliconius*, have been intensively analyzed for their elemental configuration [2,22,23], and it has been shown that three symmetry systems are developmentally equivalent, based on the observation that they can fuse together in some butterflies. As noted by Nijhout (1991) [2], Süffert (1929) [24] and Henke (1933) [25] proposed that the successive addition of such symmetry systems in wings might have occurred during the evolution of Lepidopteran insects.; these symmetry systems in a wing are considered “homologous” (sensu Nijhout (1991) [2]) to one another. Here, the term “homologous” or “homology” means that symmetry systems are likely developmentally similar at least partially, judging from morphological similarity. Furthermore, Süffert (1929) [24] noted that symmetry systems are hierarchically constructed in certain moths. However, the implications of this finding have not been explored. In butterflies, the three symmetry systems are often elaborated independently, which may obscure their homology. Nevertheless, the relationships among the symmetry systems in the nymphalid groundplan are of high importance for understanding the construction of butterfly wing color patterns.

In this paper, I examine the actual color patterns of butterflies, focusing on relationships among the color pattern systems of nymphalid hindwings. Here, important cases that show essential morphological and spatial relationships between symmetry systems are presented. Methodologically, this study is primarily aimed at identifying elements based on homology, but the overall identification results are further conceptually restructured from the viewpoints of positional relationships, hierarchy, and self-similarity. Self-similar structures (nested configurations of two (or more) systems that resemble each other) are one of the bases of fractal geometry [26]. It has been known that in the border symmetry system, border ocelli and their corresponding PFEs are self-similar (i.e., the self-similarity rule) [10] and that the discal and central symmetry systems may be self-similar [12]. Here, I examined whether this concept may be applicable not only to the relationship between border ocelli and parafocal elements, but also to the entire nymphalid color pattern. Extrapolation of this concept also leads to a model of how a symmetry system becomes asymmetric in morphology. This process of symmetry breaking may be a basis of color pattern diversity in butterflies.

## 2. Materials and Methods

### 2.1. Butterfly Specimens and Images

The butterfly specimens came from the author’s personal collection, except for *Callicore cajetani*, for which an image was kindly supplied by K. Hasuike. Species that illustrate a given point of discussion well were selected from the entire repertoire of nymphalid butterflies. Representative wings from species mostly belonging to the genera *Neope*, *Symbrenthia*, *Diaethria*, *Agrias*, and *Callicore* were included. However, the analyses conducted herein may not comprehensively cover the entire group of butterflies to which the representative species belong. All specimens that were analyzed belong to the family Nymphalidae. This study focused on the hindwings, but some forewings were also presented. 

To produce figures, images of the butterfly specimens were subjected to editing using Adobe Photoshop Elements 2019. To be consistent throughout the figures, the wing base was placed to the left and the outer margin to the right. Similarly, the wings were presented with similar sizes for comparison. Some wing images that were included in previous studies [9,12] were also included in this study but were discussed based on different aspects of color pattern analyses.

### 2.2. Color Pattern Analysis Strategies: Homology Search and Relative Location

As in the previous studies on the nymphalid groundplan [2,3,4,8,9,10,12], the procedures for the identification of color pattern elements were based on “homology”. The homology between different symmetry systems on the same wing surface and between closely related species was examined. The discal spot was often identified first because this is one of the most important steps for decoding color patterns. Equally important was the identification of border ocelli of the border symmetry system because they are often elaborated.

The relative locations of symmetry systems in relation to the discal spot were also considered important, in addition to homology. For example, the border and basal symmetry systems are always located distal and proximal to the discal spot (and hence the central symmetry system), respectively, although there are cases where the central symmetry system is not expressed. The discal spot is always located between the distal and proximal bands of the central symmetry system (dBC and pBC), provided that these bands are expressed [12]. The location of the discal spot is considered absolute (not relative) in relation to other systems because it is expressed in the potential DS area that is determined by wing veins [12].

### 2.3. Some Considerations in Elemental Identification

Regarding the process of the identification of elements, there are some points to be mentioned in this study. First, in addition to the discal spot identified in modern versions of the nymphalid groundplan, Schwanwitsch (1924) [3] identified a second discal spot (DII or D^2^) located in the discal cell, and the discal spot per se was referred to as DI or D^1^. DII should be located proximal to the proximal band of the central symmetry system (pBC), in accordance with the relative locations of systems in the nymphalid groundplan. In Süffert (1927) [4] and Nijhout (1991) [2], DII is considered the band of the basal symmetry system (BB). However, DII is considered a serial homolog of DI, and the basal symmetry system was essentially taken over by DII in some nymphalid butterflies, according to molecular and comparative studies [19,20,21]. This issue requires additional studies, and the author speculates that DII and the basal symmetry system are different entities, like the discal and central symmetry systems [12]. The present study tentatively follows Nijhout (1991) [2], but DII is also discussed as necessary in the present study. It appears that DII is mostly restricted within the discal cell, whereas the basal symmetry system can likely extend to anterior and posterior sides of the discal cell. Moreover, dislocation of the proximal band of the central symmetry system (pBC) and its alignment with DII or with the band of the basal symmetry system (BB) often occurs, which is called pierellization [27]. Thus, the distinction between pBC and BB requires very careful analysis.

Otaki (2020) [12] placed importance on the absolute locations of the potential DS area defined by wing veins (this is the absolute location of the discal symmetry system) and the relative locations of other elements in relation to the potential DS area. However, Schwanwitsch (1956) [28] did not appear to do so; both DI and DII were found to be sandwiched by dBC and pBC in the elemental identification of *Agrias claudina*. Similarly, Otaki (2020) [12] stressed that the discal spot (DI) should always be sandwiched by dBC and pBC if one places importance on the absolute location of the potential DS area (and hence the discal spot) and the relative locations of other elements in relation to the potential DS area. However, both of these bands of the central symmetry system were shown to be located proximal to the discal spot (DI) in the elemental identification of *Diaethria meridionalis* in Schwanwitsch (1956) [28]. Cases from both *Agrias* and *Diaethria* were examined because of their importance in understanding the formation of the color patterns of nymphalid butterflies in the previous study [12] and in the present analysis.

Second, the wing root band is depicted in the nymphalid groundplan of both Schwanwitsch (1924) [3] and Süffert (1927) [4] but is omitted by Nijhout (1991, 2001) [2,8]. The reason for this omission is as follows: “This pattern element occurs in some moths but is rare or absent in the butterflies” (p. 25) [2]. Otaki (2012) [9] reintroduced the wing root band in the nymphalid groundplan, but its definition has been ambiguous. In the present study, the wing root band was considered a band of the wing root band system, which is likely a half-symmetry system.

Third, a small white dot is usually located at the center of the core disk of a border ocellus. In less frequent cases, a white spot is present without a core disk, and a core disk without a white spot at the center is not particularly rare. In most cases, the white spot likely corresponds to the location of the organizer of that border ocellus. Interestingly, the white spot can behave independently of other parts of the border ocellus [29]. Nevertheless, the presence of a white spot even without the core disk is considered a mark of the center of the border ocellus in the present study.

Fourth, the concept of a secondary symmetry system that has been discussed in Nijhout (1991) [2] is taken into account in homology identification in the present study. Because this discussion is of superb quality, it is quoted below:

A significant problem with a simple diffusion gradient model for central symmetry system determination is that in some species the bands of the central symmetry system are themselves symmetry systems. … The simplest hypothesis is that in secondary symmetry systems the band itself has become a source of pattern determination. … Perhaps the hierarchy of symmetry systems (central symmetry system, flanked by basal and ocellar symmetry systems, with every band developed into a secondary symmetry system) evolved by a succession of steps like those hypothesized above. If this interpretation of pattern evolution is correct, it leads to the following generalization: Each contour on a gradient can be interpreted in two ways, as the prospective site of pigment synthesis and as the prospective site for a new source for pattern determination.

(pp. 232–233) [2]

The present study is designed to demonstrate the concept of “the secondary symmetry systems” above in nymphalid butterflies and to expand its consequences. The fractal geometry of the nymphalid groundplan might have been conjectured by Nijhout (1991) [2], although its important consequences in understanding the nymphalid groundplan were not discussed prior to the present study. Mechanistically, the induction model for positional information [10,13,17,30,31,32] is consistent with the concept of the secondary symmetry systems.

Fifth, recent examples of homology searches for elemental identification in butterfly wings have also been found elsewhere [9,12,33,34,35,36,37]. Among these cases, the genus *Cethosia*, in which white bands are present not only between elements but also between subelements within a symmetry system, is particularly interesting [9]. In the present study, detailed analyses of white bands were not performed, and these bands are a potential topic for future studies.

Finally, the assumption of homologous relationship among the symmetry systems has been widely accepted in many studies of color patterns as noted above [2,3,4,5,8,9,10,11,12,23,24,25,27,28,33,34,35,36,37,38,39], which is also supported by the concept of the secondary symmetry systems [2]. However, I acknowledge that it is difficult to completely exclude an alternative possibility that morphological similarities among the symmetry systems evolved via convergence from very different evolutionary origins. In the present study, this alternative possibility was not taken into account.

### 2.4. Beyond Elemental Identification

After elemental identification, possible relationships among symmetry systems were looked for from the viewpoints of positional relationships, hierarchy, and self-similarity. These viewpoints do not reject high degrees of developmental independence of each symmetry system. The morphological analysis was performed without considering any molecular biological data. This standpoint is necessary to eliminate potential bias at the first stage of morphological analysis. Molecular biological data were then discussed in light of the present results. 

## 3. Results

### 3.1. Three Symmetry Systems Are Morphologically Equivalent in the Genus Neope

Here, the three major symmetry systems were morphologically compared on the same wing surface. The genus *Neope* was of particular interest in this context. In *Neope bremeri* (Figure 2a), the basal symmetry system has four circular “eyespot-like” structures, which are similar to border ocelli lacking the core disk inside. The central symmetry system is a vertically elongated wide band extending from the anterior to posterior. In most anterior portion, there is a loop connection between the distal and proximal bands of the central symmetry system (dBC and pBC) (also in other *Neope* species), making it possible to consider the entire central symmetry system to be a large deformed eyespot-like structure.

In *Neope niphonica* (Figure 2b) and *Neope goschkevitschii* (Figure 2c), the central symmetry system in each wing compartment appears to be more independent, forming eyespot-like structures in each compartment. It is clear in these two species (also seen in other *Neope* species) that the color patterns of the central symmetry system are the same as those of the border ocelli on the same wing, although the core elements (central band) of the central symmetry system (cBC) are not as dark as those of the border ocelli. In the individual of *Neope goschkevitschii* shown in the figure below (Figure 2c), the most posterior ocellus of the border symmetry system is small and lacks a core disk, similar to those of the basal symmetry system. In *Neope muirheadi* (Figure 2d), the basal symmetry system includes three or four eyespot-like structures, again lacking a core disk. In *Neope yama* (Figure 2e), the basal symmetry system is conspicuous as a fusion of three consecutive eyespot-like structures, forming the distal and proximal bands of the basal symmetry system (dBB and pBB), although the central band (cBB) does not exist. An additional spot of the basal symmetry system likely exists in the CuA_2_ compartment.

In these five species of *Neope*, the anteroposterior positioning of the spots of all three symmetry systems forms a gentle S-shaped curve, suggesting the wing-wide coordination of the elemental configuration. The most posterior spot of the basal symmetry system is located in the CuA_2_ compartment and close to the wing base, but its identity is likely correct because of its structure, coloration, and the S-shape positioning of spots in all three systems. The discal spot (DS) is expressed as simple narrow bands on the discal cross vein sandwiched by the distal and proximal bands of the central symmetry system (dBC and pBC). A DII spot should resemble this DS if it exists, but it is not expressed in these *Neope* species, suggesting that DII and the basal symmetry system are different systems.

Taken together, the central symmetry system can be expressed as a large vertically elongated circular structure, but it can be divided into small parts by the wing veins. In this context, a single unit is similar to an eyespot. A similar example of serial eyespot-like structures is the central symmetry system found in *Mantaria maculata*, as described by Nijhout (1991) (p. 31) [2]. The basal symmetry system can also be expressed as spots or bands. In addition to structural similarity, the coloration of the system is similar among the three symmetry systems. In many *Neope* species, yellow coloration is observed between the core disk and outer black ring in border ocelli, and yellow coloration is similarly located in other systems. Therefore, the three symmetry systems are likely morphologically (and, hence, developmentally) equivalent (i.e., homologous) based on the structures and coloration of elements of the genus *Neope*. The coloration of the field space between subelements, in which different systems may be colored the same, may be called the field coloration rule.

### 3.2. Diverse Arrangements of the Border Symmetry Systems in the Genus Symbrenthia

**Figure 3:**
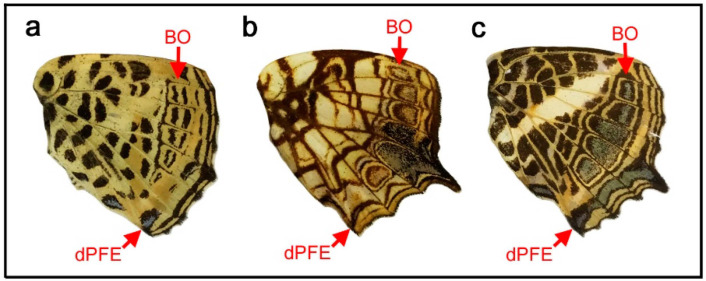
Diversity of band arrangements of the border symmetry system in the genus *Symbrentha*. BO: border ocelli, dPFE: distal parafocal element. (**a**) *Symbrenthia leopard*. (**b**) *Symbrenthia hippoclus*. (**c**) *Symbrenthia hypselis*.

Similar to the diverse arrangements of the central and basal symmetry systems in the genus *Neope* described above, the genus *Symbrenthia* shows flexible expression of the border symmetry system. In *Symbrenthia leopard* (Figure 3a), border ocelli (BO) are expressed as parallel lines. The areas inside these “eyespots” are blank; that is, they are continuous with the background area. This is one of the examples giving rise to the proposal of the binary color rule (or simply, the binary rule) in a previous study [16]. In this species, the distal parafocal element (dPFE) is expressed as a collection of lines, but two of them are expressed as a pair of parallel lines. The most posterior is filled with a bluish structural color, a feature of dPFEs in Nymphalidae. In *Symbrenthia hippoclus* (Figure 3b), the border ocelli are serially positioned, and their central area remains blank except in two ocelli with a central area filled with black color and a bluish structural color. The most posterior dPFE fragment is a pair of parallel lines, between which is a blank space. In *Symbrenthia hypselis* (Figure 3c), most border ocelli are filled with a bluish structural color. Again, the dPFE is filled with the same bluish structural color, but only at the posterior. In contrast, the dPFE at the anterior segment is expressed as a simple band.

In summary, in *Symbrenthia*, border ocelli can be expressed not only as eyespots, but also as line or band elements. When expressed as broken lines, the area between these elements is background. When this background area is enclosed, bluish structural coloration tends to develop. Interestingly, these characteristics are applicable not only to border ocelli but also to dPFE, suggesting their self-similar relationship.

### 3.3. The Discal Symmetry System (Discal Spot) May Be Expressed as a Circular Eyespot-Like Structure

In the previous study, the discal spot has been shown to be expressed in diverse structures and considered as a symmetry system [12]. Although eyespot-like structures of the discal symmetry system have been known well in Saturniidae moths and some species of Riodinidae butterflies, such circular structures are rare in Nymphalidae. Since circular eyespot-like structures of the three major symmetry systems were shown in *Neope* (Section 3.1), it is important to examine if the discal spots in Nymphalidae can form such a structure. 

**Figure 4:**
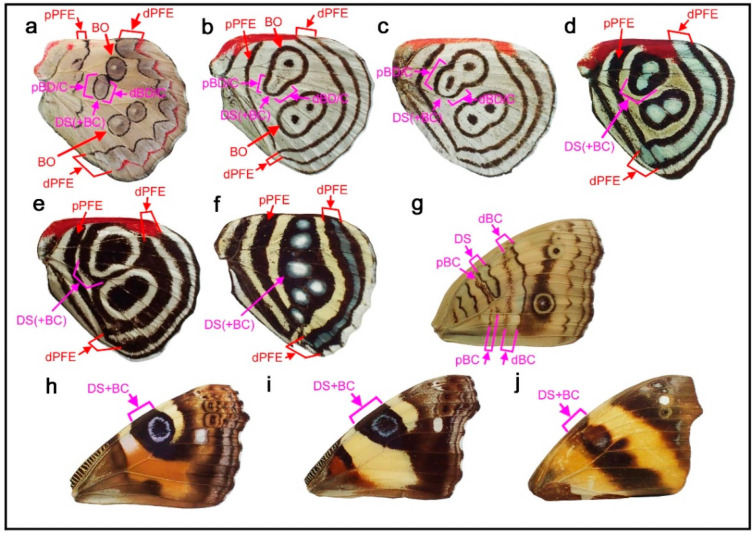
Circular patterns of the discal spot in the family Nymphalidae. (**a**) *Diaethria bacchis*. (**b**,**c**) *Diaethria neglecta*. (**d**) *Diaethria* (*Catacore*) *kolyma*. (**e**) *Diaethria clymena*. (**f**) *Callicore hesperis*. (**g**) *Junonia almana*. (**h**) *Vanessa* (*Bassaris*) *gonerilla*. (**i**) *Vanessa* (*Bassaris*) *itea*. (**j**) *Epiphile dinora*.

It appears that *Diaethria bacchis* (Figure 4a) has a relatively large circular eyespot-like discal spot in the potential DS area (defined by the wing veins) in the ventral hindwing. Indeed, this discal spot is structurally similar to border ocelli (BO) that are located close to the discal spot. Interestingly, the thick and doubled pPFE is unambiguously identifiable because of a silver structural color inside. Large BO and pPFE are clearly observed, but there is no band of the central symmetry system, which should sandwich the discal spot [12]. Thus, an alternative identification is that only the central portion of the discal spot identified above is the actual discal spot, which is surrounded by the distal and proximal bands of the central symmetry system (dBC and pBC). In other words, the eyespot-like structure in the potential DS area may be a fusion of the discal and central symmetry systems. Following this latter interpretation, in an individual of *Diaethria neglecta* (Figure 4b), the potential DS area contains a vanishingly weak bar-like structure at the center, which may be the discal spot. There is nothing unique about this discal spot itself in its structure and location, and this discal spot is surrounded by black bands, which likely represent the distal and proximal bands of the central symmetry system (dBC and pBC). They are connected together at the posterior side, forming a U-shape. They are further connected with the anterior border ocelli (BO), but the connection points can be inferred by inflection points of the bands. An alternative identification is that these bands and the bar-like structure inside all belong to the discal symmetry system, and a large eyespot-like structure is attained by the discal symmetry system; however, in that case, the central symmetry system is not expressed. In a different individual of the same species (Figure 4c), the bar-like central structure is thicker, and the surrounding bands are more circular than those in the previous individual. Another interesting observation in this species is that dPFE is likely “doubled”, forming two bands.

As an extension, *Diaethria* (*Catacore*) *kolyma* (Figure 4d) is also interesting. It has an eyespot-like structure in the potential DS area, which may be the discal spot or a complex of the discal and central symmetry systems. The doubled dPFE is filled with a bluish structural color. In *Diaethria clymena* (Figure 4e), black bands are very thick, and the discal spot or a complex of the discal and central symmetry systems is indeed slightly larger than the potential DS area. In *Callicore hesperis* (Figure 4f), an eyespot-like structure is present in the potential DS area.

In these cases above, a distinction between the discal and central symmetry systems is not possible. However, the discal symmetry system appears to be able to form a circular eyespot-like structure at least as a fusion with the central symmetry system. In the ventral forewing of *Junonia almana* (Figure 4g), the distal and proximal bands of the central symmetry system (dBC and pBC) are clearly observed, together with the elongated U-shaped discal spot. The ventral forewings of *Vanessa* (*Bassaris*) *gonerilla* (Figure 4h), *Vanessa* (*Bassaris*) *itea* (Figure 4i), and *Epiphile dinora* (Figure 4j) has a circular discal spot, although this discal spot is completely enclosed with the bands of the central symmetry system. Therefore, it is reasonable to conclude that the discal symmetry system has an ability to express a circular eyespot-like structure, like other major symmetry systems.

### 3.4. Hierarchical Arrangement of the Symmetry Systems in the Genus Agrias

**Figure 5:**
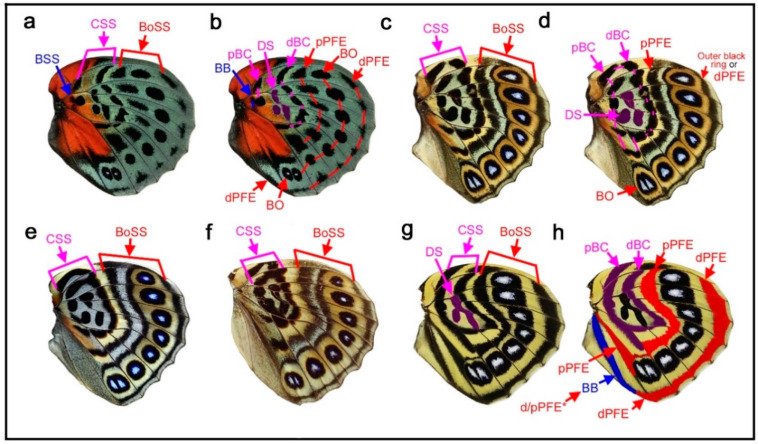
Diversity of the genus *Agrias*. See Figure 1b for abbreviations. (**a**,**b**) *Agrias hewitsonius*. These two wing images are identical except for the annotations. (**c**,**d**) *Agrias claudina*. These two wing images are identical except for the annotations. (**e**) *Agrias claudina*. This is a different individual from that in c and d. (**f**) *Agrias sahlkei*. (**g**,**h**) *Agrias amydon*. These two wing images are identical except for the annotations. An alternative identification of BB is indicated as d/pPFE*.

Given the above results showing that all four symmetry systems have developmentally equivalent potential to form various structures, including circular ones, four intriguing species of the genus *Agrias* were examined herein to understand the relationships among the symmetry systems. In *Agrias hewitsonius* (Figure 5a,b), there are many spots, but the identification of border ocelli (BO) is easy because the posterior two ocelli exhibit foci, and the others are also circular spots. Accordingly, distal and proximal parafocal elements (dPFE and pPFE) are easily identified. There does not seem to be submarginal or marginal band (SMB or MB), and the dPFE is located close to the wing edge. Additionally, identification of the discal spot (the discal symmetry system) is not difficult because it is located in the potential DS area defined by the wing veins [12]. It seems that all three spots in the potential DS area belong to the discal spots, which is not overly peculiar considering a previous study on discal spot diversity [12]. The discal spots are surrounded by the distal and proximal bands of the central symmetry system (dBC and pBC). The most basal spots in the discal cell likely represent the band of the basal symmetry system (BB). Thus, the discal symmetry system is clearly nested within the surrounding central symmetry system. The red area at the wing base is enigmatic but may be considered bold background coloration.

In *Agrias claudina* (Figure 5c,d), the discal spots are identifiable based on the potential DS area, and they are enclosed by dBC and pBC spots. This enclosure is clearer than that in the previous species. The enclosed area (i.e., the intrasystem background or interelement background) is colored light blue. The border symmetry system includes many eyespots; outer black rings (or dPFEs) are present without the marginal band system; and pPFE runs parallel with dBC, forming an arc. The space between dPFE and BO is the intrasystem background (interelement background) and is colored light blue. This coloration is similar to that of another type of intrasystem background of the central symmetry system on this wing, as mentioned above. In contrast, the gap between the border and the central symmetry system (i.e., intersystem background) is colored yellow. It is noteworthy that the space between the eyespot core disks and the outer black rings is also colored yellow. Although this “space” (or similar ones) is often considered a distinct eyespot “ring” in the literature, it is more appropriately considered the intrasystem background (intraelement or intersubelement background) based on the binary rule [12,16].

The central symmetry system and the border symmetry system are both filled with light blue coloration, and they can thus be considered homologous. Additionally, they are separated by yellow areas. An additional yellow area within the border symmetry system likely indicates that the core disk and the outer black ring within the same eyespot can be considered separate. Therefore, the above light blue and yellow color assignments indicate the hierarchical and self-similar nature of the nymphalid color patterns. That is, a core-paracore relationship holds true between the central and border symmetry system and between the core disk and the outer black ring (or parafocal elements) within the border symmetry system, as indicated by field coloration. The finding that the background spaces of homologous elements are filled with similar colors follows the field coloration rule (Section 3.1).

Similar yellow coloration is present at the wing base, although no spots are associated with this background coloration. It may be speculated that the basal symmetry system is not expressed and that only the intrasystem background associated with the basal symmetry system is expressed. If so, the red background around the wing base observed in the previous species may also be associated with the basal symmetry system. A different individual of the same species showed more complete enclosure of the discal spots by the central symmetry system (Figure 5e), confirming the identifications discussed above. Furthermore, in *Agrias sahlkei* (Figure 5f), the enclosing circle is smaller.

Then, the case of *Agrias amydon* (Figure 5g,h) was examined. The discal spots were identified first within the discal cell (Figure 5g). Although the discal spots in this species are somewhat irregular, they are nonetheless located in the potential DS area. On their distal and proximal sides, the discal spots are sandwiched between dBC and pBC (Figure 5h). BO were easily identified. Similar to the previous species, pPFE encloses the entire central symmetry system. The thick band at the wing margin probably does not represent the marginal system because it is too wide for the bands of the marginal band system, and no marginal band system is expressed in the two previous species of the same genus. Instead, this band is a likely dPFE. Additionally, dPFE encloses the entire central and border symmetry system, but more appropriately, dPFE is connected with a possible basal band at the tornus, although the identification of the basal band (BB) here is tentative. This merging of the two elements may suggest that the border and basal symmetry systems are continuous in origin but physically divided because the entire system does not spatially fit the wing tissue.

Taken together, these examples from *Agrias* demonstrate the self-similar nature of the systems on the basis of two characteristics: the hierarchical spatial arrangements of elements and the hierarchical color arrangements of the intersystem and intrasystem background on the hindwings.

### 3.5. The Border Symmetry System Can Occupy Most Parts of the Hindwing in the Genus Callicore

**Figure 6:**
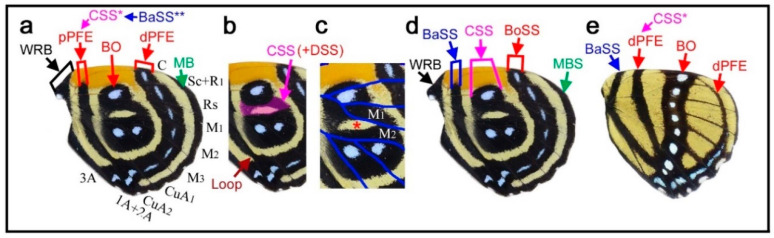
Analysis of eyespot-like structures at the center of the wing in *Callicore cynosura* and *Callicore cajetani*. See Figure 1b for abbreviations. (**a**) *Callicore cynosura* with the preferred element identification. Alternative identifications of pPFE are indicated as CSS* and BaSS** (not preferred). The names of wing compartments are indicated. (**b**) The central area of the wing shown in a. The possible central symmetry system (CSS) around the potential DS area is highlighted in pink. Looping connections between the distal and proximal parafocal elements are indicated. (**c**) Further magnification of the central area of the wing shown in a. Wing veins are indicated in blue. An asterisk at the center of the wing indicates the location of the potential discal spot. The compartments M_1_ and M_2_ (assuming the presence of the discalis cross vein) are indicated. (**d**) *Callicore cynosura* with alternative identifications (not preferred). This wing image is identical to a except for the identifications of elements. (**e**) *Callicore cajetani*. An alternative identification of pPFE is indicated as CSS* (not preferred).

Here, several species of the genus *Callicore*, in which distinct eyespots or eyespot-like structures are located proximally, were analyzed. The first species, *Callicore cynosura* (Figure 6a–d), has three eyespot-like structures that are fused together, containing bluish structural color at the center. These eyespot-like structures occupy the physical center of the wing. In the previous analysis of the related species (Figure 4a–f), these eyespot-like structures are considered border ocelli (BO) without any consideration of other possibilities, and the BO identifications are likely correct (as shown below). However, because not only the border symmetry system but also the central, basal, and discal symmetry systems can potentially form eyespot-like structures, as discussed previously (Section 3.1 and Section 3.3), it is important to logically assure that these eyespot-like structures belong to the border symmetry system.

As default, the eyespot-like structures that occupy the central area of the wing are considered border ocelli (BO), and accordingly, pPFE and dPFE are assigned (Figure 6a), despite their exceptional location at the center of the wing for the following reasons. First, they are structurally typical border ocelli of nymphalid butterflies (although they are only core disks in this species), with the bluish structural color at the focus. Second, these eyespot-like structures are located within the compartments where border ocelli are supposed to be located despite their proximal dislocations; they are not located in the discal cell. Third, these eyespot-like structures are associated with a band that is typical of a distal parafocal element (dPFE) in nymphalid butterflies; it contains the bluish structural color inside.

The assignment of pPFE is not straightforward. This band was considered the dislocated central symmetry system by Schwanwitsch (1956) [28] (see discussion in the previous study [12]). Importantly, the potential DS area defined by the wing veins has a blank background area with an elongated oval shape, and the elongated oval region is likely surrounded by its own black ring (Figure 6b,c). The discal spot may not be expressed (but the potential DS area nevertheless remains blank), and its surrounding black region may be considered the central symmetry system. Alternatively, this black region may also have a contribution from the discal spot if it is expressed. 

In contrast, a very different set of identifications is still possible (although not preferred); the three large eyespot-like structures may belong to the central symmetry system simply because they surround the potential DS area at the center of the wing (Figure 6d). Even in this case, there would be no conflicting identification afterward; all three major symmetry systems could be identified in addition to the two peripheral systems.

Although no additional information was found in this wing itself, this problem was solved simply by referring to another species of the same genus, *Callicore cajetani* (Figure 6e). In this species, a series of white spots are embedded within a black band. On the basis of their locations (distal to the potential DS area), these spots are unquestionably BO of the border symmetry system. Distal to these BO, a dPFE consisting of a band forming an arc is located. The identification of this dPFE band is also unequivocal; a posterior part of this band is filled with a bluish structural color, which is a general feature of dPFEs. Therefore, the identifications presented in Figure 6a are preferred to those in Figure 6d for *Callicore cynosura*.

The band proximal to the band of BO in *Callicore cajetani* (Figure 6e) is more difficult to identify; it may be a proximal parafocal element (pPFE), but it may be a distal band of the central symmetry system (dBC) because this band spans the potential DS area in this species. However, the identification of pPFE is preferred because this band in *Callicore cajetani* is likely homologous to a pPFE of *Callicore cynosura* (Figure 6a). This identification of pPFE is consistent with those in *Diaethria* (Figure 4). The further proximal band is more difficult to identify (tentatively annotated as BaSS in Figure 6e), but will be discussed in subsequent sections (Section 3.6 and Section 3.7).

Returning to the first species, *Callicore cynosura*, with the final identifications (Figure 6a), there is a marginal band (MB) but no submarginal band (SMB). The proximal wing margin is also decorated by a black band in this species, which may be an extension of the wing root band (WRB). The wing root band (WRB) is connected to MB and to dPFE near the tornus. In addition, pPFE is connected to dPFE at the tornus (Figure 6b), which justifies this identification of pPFE. This identification also satisfies the distal elaboration rule (see below).

In summary, in *Callicore cynosura* and related species (below), the central symmetry system is, if expressed, likely located in the potential DS area defined by wing veins (at the physical center of the hindwing), and the border symmetry system surrounds the central symmetry system. Within the border symmetry system, the border ocelli are surrounded by bands of dPFE and pPFE; they are connected at the tornus, suggesting that they are a single entity in origin. This hierarchical configuration is similar to that of *Agrias* (Section 3.4), suggesting the self-similar relationship among the symmetry systems.

### 3.6. The Potential DS Area Is Blank in Many Species of the Genus Callicore

**Figure 7:**
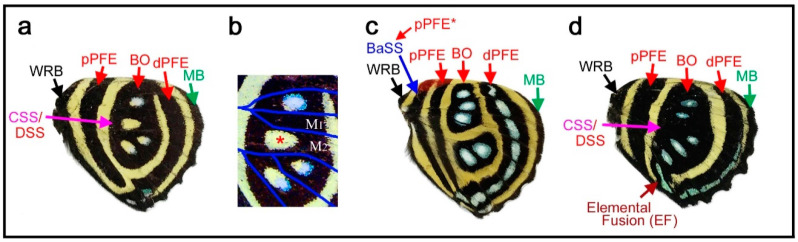
Three *Callicore* species with various patterns of the potential DS area. (**a**) *Callicore hydaspes*. (**b**) The discal area of the wing shown in a. An asterisk indicates the location of the potential discal spot. The compartments M_1_ and M_2_ (assuming the presence of the discalis cross vein) are indicated. (**c**) *Callicore pygas*. An alternative identification is shown as pPFE*. (**d**) *Callicore lyca*.

A color pattern similar to the above case (*Callicore cynosura*) was found in *Callicore hydaspes* (Figure 7a,b). In this species, there are four eyespot-like structures that merge together, each of which has a blank region at the center. However, a magnified image revealed that three of these structures contain a bluish structural color and that the blank region located in the potential DS area does not contain anything like this (Figure 7b), indicating a difference in the blank DS area from the rest. 

**Figure 8:**
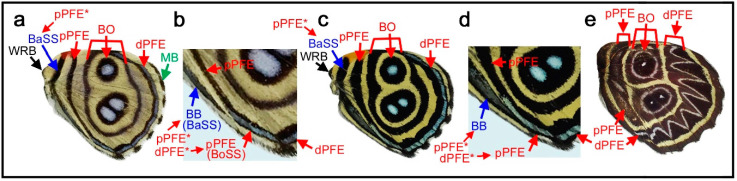
Three *Callicore* species with more complex patterns. Alternative identifications are indicated by asterisks. (**a**) *Callicore hystaspes*. (**b**) The tornus of the wing shown in a. (**c**) *Callicore eunomia*. (**d**) The tornus of the wing shown in c. (**e**) *Callicore sorana*.

In *Callicore pygas* (Figure 7c), the border ocelli are divided into anterior and posterior clusters by the potential DS area. These cases of *Callicore pygas* (Figure 7c) and *Callicore cynosura* (Figure 6a) are not rare. In fact, many other *Callicore* species have the blank DS area (see Figure 8 for additional examples). In addition, the previous study on the discal symmetry system showed that the potential DS area is not completely invaded by the border symmetry system [12]. This finding may be summarized as the DS area protection rule; the potential DS area tends to be protected from the potential invasion of the border symmetry system. 

In *Callicore lyca* (Figure 7d), the potential DS area is painted black and does not include a blank region or bluish structural color. Other compartments include bluish border ocelli (BO). At first glance, this may seem to be an exception of the DS protection rule. However, the black color in the potential DS area may be contribution of the central and/or discal symmetry system, because both the central and discal symmetry system are allowed to be present in the potential DS area. Interestingly, all bands are connected at the posterior near the tornus, but the bluish areas of dPFE and the border ocelli fuse together, suggesting the equivalence of their developmental mechanisms, in accordance with the self-similarity rule of the border symmetry system [8]. 

### 3.7. More Complex Color Patterns in the Genus Callicore: Basal Band or Doubled pPFE

Color patterns that are more complex than the previously described patterns can be seen in *Callicore hystaspes* (Figure 8a,b) and *Callicore eunomia* (Figure 8c,d). The anterior and posterior eyespots are physically divided by the potential DS area in these species, following the DS area protection rule. Notably, in these species, an additional band is present near the wing base. This band may be the basal band (BB), because the central symmetry system is not expressed and the proximal half of the wing is “invaded” by the border symmetry system in this genus. However, this identification is tentative because it may also be an additional band of pPFE (see below). 

The potential existence of pPFE in *Diaethria* is discussed in Figure 4 (Section 3.3). Here, pPFE and dPFE are connected together at the tornus, although their boundary is not clear. The band tentatively identified as BB is not directly connected in *Callicore hystaspes* (Figure 8a,b). In *Callicore eunomia* (Figure 8c,d), dPFE and pPFE are completely connected, and the band tentatively identified as the BB contact dPFE. These findings indicate the circular nature of the parafocal elements, as shown in *Agrias* (Section 3.4) and other *Callicore* and its related species (Section 3.3 and Section 3.5). This connection between dPFE and pPFE justifies the identification of these bands. One concern is that the band tentatively identified as BB also contains a bluish structural color inside, both in *Callicore hystaspes* (Figure 8a,b) and *Callicore eunomia* (Figure 8c,d); these bands may also belong to pPFE. In that case, pPFE is expressed as a double band. In *Callicore sorana* (Figure 8e), both dPFE and pPFE are thick and contain a silver structural color inside, supporting the idea that pPFE exists in this and related genera. The thick pPFE containing the silver structural color in this species may be considered a “fusion” of the doubled pPFE in the previous two species. Since dPFE is doubled clearly in *Diaethria* (Figure 4), the doubled pPFE in *Diaethria* and *Callicore* is a likely possibility.

### 3.8. The Distal Elaboration Rule and Its Exceptions

As seen in the color pattern analyses of the border symmetry system described above, dPFE is generally well expressed (often with bluish structural color inside), whereas pPFE is not. The same tendency is observed in the central symmetry system: dBC is often better elaborated than pBC. The applicability of this tendency to the basal symmetry system is uncertain because the basal symmetry system is not fully elaborated in most cases. At the wing-wide level, the border and basal symmetry systems can be considered the paracore elements of the supersymmetry system, and the distal system (the border symmetry system) is certainly better elaborated than the proximal system (the basal symmetry system). These characteristics may be summarized as the distal elaboration rule for a pair of paracore elements. Thus, the distal elaboration rule holds true in a symmetry system and in the wing-wide supersymmetry system.

**Figure 9:**
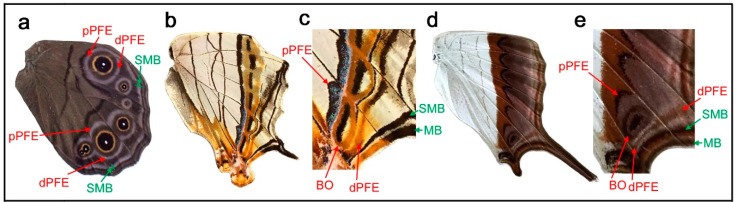
Exceptional cases of the distal elaboration rule. (**a**) *Lethe diana*. (**b**) *Cyrestis thyodamas*. (**c**) The distal area of the wing shown in (**b**). (**d**) *Marpesia coresia*. (**e**) The distal area of the wing shown in d.

Although most species in Nymphalidae follow the distal elaboration rule, there are some exceptional cases. In some species of the genus *Lethe*, both dPFE and pPFE contain a bluish or silver structural color. Here, *Lethe diana* (Figure 9a) is shown. Notably, they are connected to completely encircle some border ocelli like a part of the eyespot ring. In this individual, pPFE may be slightly thicker than dPFE in the posterior portion of the wing. A more extreme exception is the border symmetry system of *Cyrestis thyodamas* (Figure 9b). In this species, pPFE contains bluish structural color inside, but dPFE does not (Figure 9c). As discussed above, the bluish structural color is a landmark feature that is often found in dPFEs in many nymphalid species. A less clear case is seen in the border symmetry system of *Marpesia coresia* (Figure 9d), where pPFE is wider than dPFE (Figure 9e).

## 4. Discussion

### 4.1. Identification of Elements Based on the Nymphalid Groundplan

In this paper, the color patterns of representative nymphalid butterflies were analyzed based on homology searches, assuming that actual color patterns can be understood as modifications of the nymphalid groundplan. The process of the identification of elements (i.e., which band or spot belongs to which system and element) was not always straightforward. To identify elements, similar but different species were compared, and the relative locations of systems in reference to the potential DS area were taken into account. Although the color patterns are sometimes very complex and there is often no information for performing identification within a single wing, sufficient clues for identification were obtained in most cases. This success even in complex nymphalid butterflies such as *Neope*, *Symbrenthia*, *Diaethria*, *Agrias*, and *Callicore* verifies that the current nymphalid groundplan is correctly formulated. The important question asked here is how the elemental configuration can be (and cannot be) produced and modified. In this sense, this paper aims to understand the developmental potential and constraints of the nymphalid groundplan as a system.

A self-similar configuration of the nymphalid groundplan proposed in this study is not readily discernable in most butterflies. This is likely because each system is extensively modified independently. In this sense, this study could not provide a predictive model. Rather, this study proposed possible relationships among the symmetry systems behind extremely diverse color patterns. Importantly, modifications likely follow a set of color pattern rules proposed here and in previous studies [10,32].

### 4.2. Developmental Equivalence of the Symmetry Systems

The central symmetry system, which is often expressed as bands, can form eyespot-like structures in *Neope* (Figure 2). The entire central symmetry system may be a single large circular structure, as indicated by the loop connections between distal and proximal bands (dBC and pBC) at both the anterior and posterior ends of the system. Furthermore, the entire central symmetry system can be divided by wing veins to produce an array of individual eyespot-like structures. The basal symmetry system is not well developed in comparison to other symmetry systems, but the basal symmetry system also sometimes contains several bands or spots in *Neope* (Figure 2), as shown in a previous study [12]. In *Symbrenthia*, the border symmetry system, which is often expressed as eyespots, can be expressed as bands (Figure 3). Therefore, all three major systems potentially have the ability to express eyespot-like structures or bands; they are likely developmentally equivalent (or “homologous”). Additionally, the discal symmetry system can form circular patterns (Figure 4). It should be noted that this simple but important finding is not readily discernable from an illustration of the nymphalid groundplan [2,3,4,8,9,10,12], except for the simplified schematic representation (Figure 1) (also see [9]). This developmental equivalence is a necessary condition for self-similarity. All three systems may originate as a large circular entity that is elongated in the anteroposterior direction, which is then divided into an array of individual spots by the wing veins.

Developmental equivalence of all symmetry systems can also be suggested by the coloration of systems. In this paper, examples are seen in *Neope*, where all three major symmetry systems have similar coloration, and in *Agrias*, where intersystem background and intrasystem background are colored similarly in the central and border symmetry systems. In a previous study [12], a similar relationship is seen in *Tanaecia trigerta*, *Euthalia lubenthina*, and *Vanessa* species, where the discal and border (and other) symmetry systems have similar coloration, and in *Tarattia lysanias*, *Dynamine aerata*, and *Morpho hecuba*, where the central and border symmetry system have similar coloration. These cases may be summarized as the field coloration rule.

Previous morphological studies have emphasized that the three symmetry systems are almost completely independent during development based on morphometric correlation analysis [2,38,39]. It is to be noted that developmental equivalence shown in this study does not necessarily contradict independence of each symmetry system. Instead, it would be more reasonable to consider that serial induction confers each system independent developmental potential, as suggested by the induction model [10,13,16,17]. Independence of each element (within the same symmetry system) is an important source of color pattern diversity. Similarly, independence of each subelement (even within a given eyespot) is likely an important source of color pattern diversity, which is called the uncoupling rule [10]. Nonetheless, there are likely some interactions between two symmetry systems at their boundaries, although such cases were not directly shown in this study.

### 4.3. Interpretations of Molecular Biological Studies

The present study suggests that the organizer of the discal symmetry system (DSS) dictates other symmetry systems (Section 4.5, Section 4.6, and Section 4.9), but there is no direct molecular evidence for this hypothesis at present. Moreover, developmental equivalence of symmetry systems presented in this paper may seem to be in conflict with molecular data at first glance, which shows that different genes are functionally expressed in different systems during development [19,20,21]. For example, *WntA* functions to form the central symmetry system [20,21], and *wingless* is expressed in the discal symmetry system [19]. *Distal-less* and *spalt* function to form border ocelli of the border symmetry system [40,41,42,43]. These molecular data are important but do not support or reject the concepts of homology and hierarchy among the symmetry systems, because there should be differentially expressed genes to modify and elaborate each system regardless of the mechanistic unity of the three systems. More importantly, the self-similar configuration based on serial induction occurs before the molecules such as *WntA* and *Distal-less* become active, as suggested by the induction model [10,13,16,17]. In other words, these molecules may simply constitute a signal for the final color pattern of a given system. 

A recent study on the CRISPR-Cas9 mosaic mutants of *Distal-less* [43] appears to indicate independence of border ocelli. Eyespot phenotypes of these mutants were simulated well without considering parafocal elements and other symmetry systems [43]. However, in this simulation, boundary conditions are important to induce eyespots, but boundary conditions in a real wing tissue may be given as interactions from other organizers. For instance, in this simulation, a sink is provided by the proximal vein, but it may be provided by the DSS organizer in a real wing tissue (Section 4.9). Unfortunately, parafocal elements have not been considered carefully in this important study [43].

Importance of boundary conditions to induce border ocelli has also been implicated by a gene transfer study [41]. Ectopically expressed *Distal-less* using a baculovirus-mediated gene transfer method can induce elemental patterns and black spots but not a full eyespot [41]. That is, where to express *Distal-less* is likely determined by surrounding signals, and *Distal-less* expression does not guarantee expression of a full eyespot. 

A conventional interpretation of the CRISPR-Cas9 mosaic mutants of *Distal-less* in *Bicyclus anynana* [43] is that eyespot centers are specified by *Distal-less*. However, an alternative interpretation based on heterochrony is that eyespot centers can be specified without *Distal-less*, because parafocal elements did not appear to be affected in other mutants in *Bicyclus anynana* [44]. In eyespot-less mutants, the eyespot center may be formed and release the earliest signal, which is for parafocal elements, but the activity of the center deteriorates quickly, and subsequent signals for eyespot rings cannot be released. In this scenario, *Distal-less* is not required for eyespot center specification, but *Distal-less* is required for making the center active longer to produce eyespots (but not parafocal elements in eyespot-less mutants of *Bicyclus anynana* [44]). Interestingly, parafocal elements as well as submarginal bands are likely affected in the CRISPR-Cas9 mosaic mutants of *Distal-less* not only in *Bicyclus anynana* [43] but also in *Junonia coenia* and *Vanessa cardui* [42].

It is interesting that parafocal elements appear to be genetically robust in *Bicyclus anynana* [44] but parafocal elements can easily be modified physiologically in many species [45,46,47]. This discrepancy is enigmatic at this point, but species in Satyrinae (including *Bicyclus anynana* and *Ypthima argus*) may have parafocal elements that are resistant against physiological treatments [48]. The genetic resistance of parafocal elements in *Bicyclus anynana* [44] is also reminiscent of the fact that physical damage at the prospective eyespot center can never be able to affect parafocal elements [49,50,51,52] in contrast to physiological treatments. This is known as the PFE paradox, which has also been explained as a heterochronic effect [16]. Unless these similar results of molecular and physical manipulations are a simple coincidence, such a heterochronic effect could be an explanation for the nearly intact parafocal elements in the eyespot-less mutants of *Bicyclus anynana* [44]. In contrast, in some CRISPR mutants [42,43], the activity of *Distal-less* may be so strong or long-lasting that parafocal elements are affected.

On the other hand, *cortex*, which is a gene that functions in cell cycle, is also known to function in color pattern formation, but its expression does not completely correspond to a particular symmetry system [53,54]. Cell cycle control is a key to change cell size and scale size in the induction model [10,31]. In any case, possible interpretations of the molecular data available at present are not singular. I stress that both molecular and morphological analyses have not been performed sufficiently to completely understand the relationships among the symmetry systems in the nymphalid groundplan.

### 4.4. Structural Similarities between Border Ocelli and Parafocal Elements

The self-similarity of the border symmetry system (between border ocelli (BO) and distal parafocal elements (dPFEs)) has been discussed briefly in a previous study [8]. Because the PFE is a paracore element of the corresponding core element (i.e., border ocellus), the morphological similarities between them indicate a self-similar relationship. In this context, many of the *Callicore* species presented in this paper are intriguing examples (Figure 4 and Figure 6, Figure 7, Figure 8). In *Callicore cynosura* (Figure 6a) and *Callicore pygas* (Figure 7c), BO and dPFE are similar in color. In the latter species, there is a white focal area at the center of both BO and dPFE surrounded by bluish structural color embedded within a black band. In these and other species of *Callicore*, the bluish structural color in dPFE forms an elongated bar-like structure that is slightly different from circular BO, but BO and dPFE fuse together in a species (Figure 7d), indicating that BO and dPFE are developmentally equivalent. Similarities in color and shape between BO and dPFE are also observed in *Symbrenthia hypselis* (Figure 3c).

Similar to the relationship between the distal and proximal bands of the central symmetry system (dBC and pBC) that are connected at the anterior and posterior ends in *Neope* (Figure 2), dPFE and pPFE are connected at the tornus or apex in some species, especially in *Callicore* (Figure 6, Figure 7 and Figure 8), indicating that these two bands are originally a single circular system.

Interestingly, one of the CRISPR-Cas9 *Distal-less* mosaic mutants shows two eyespots in a single compartment [43]. One of them shows a normal size and is located at the normal position, whereas the other is smaller and is located at the position of dPFE [43]. Because normal dPFE may be lost in that compartment, the small ectopic eyespot can be identified as dPFE. If so, this phenotype could be a genetic demonstration that PFE is developmentally equivalent to BO and thus they are self-similar.

### 4.5. The Potential DS Area May Be Protected: The DS Area Protection Rule

Although all systems likely have equivalent developmental potential, the discal symmetry system is unique in many aspects [12]. Notably, it is fixed in location at the center of the wing within the potential DS area defined by wing veins. Additionally, as seen in *Callicore* in the present study, the potential DS area appears to be “protected” from invasion of the border symmetry system (the DS area protection rule) [12]. The potential DS area in many species of *Callicore* is blank probably because of unseen activity of the discal symmetry system. To be protected, the area is to be occupied by a morphogenic signal from the DSS organizer before signals from other organizers reach this area. In other words, this uniqueness of the discal symmetry system may originate from its special status (i.e., the earliest emergence) among the symmetry systems in constructing and coordinating the wing-wide color patterns.

### 4.6. The Hierarchy of the Symmetry Systems

Importantly, the discal spot can be surrounded almost circularly by the central symmetry system, and the central symmetry system can be further similarly surrounded by the border symmetry system. The status of the basal symmetry system is less certain, but it may form a large circular structure in conjunction with the border symmetry system. This configuration is most conspicuous in *Agrias* (Figure 5) and also in *Diaethria* (Figure 4) and in *Callicore* (Figure 6, Figure 7 and Figure 8). This pattern demonstrates that the discal symmetry system (and its associated central symmetry system) is located at the center of the wing-wide color pattern and that the border and basal symmetry systems originate as a continuous single circular system that is simply physically divided in two into distal and proximal sides. The central symmetry system corresponds to a “core element” and the border and basal symmetry systems to a pair of “paracore elements” according to the core-paracore rule, like a large eyespot-like structure. Thus, these symmetry systems may collectively form a unified supersymmetry system. Accordingly, the self-similarity rule can be considered to be generalized in this study. It should also be noted that the discal symmetry system and the central symmetry system may also be self-similar [12].

In each symmetry system, the central band (cBD, cBC, cBB, or BO) is a core element that accompanies a pair of paracore elements. Thus, the core-paracore relationship is repeated hierarchically. This hierarchy is also indicated by coloration, according to the field coloration rule.

### 4.7. Evolutionary Developmental Plasticity of the Systems

The *Callicore* (and also *Diaethria*) color pattern is mostly composed of the border symmetry system in the preferred (likely more logical) identifications (for example, see Figure 6a), but its configuration is similar to the original configuration of the nymphalid groundplan with the three symmetry systems. Indeed, alternative identifications (see Figure 6d for comparison), although likely incorrect, nonetheless match the overall configuration of the nymphalid groundplan. The implication is that a single system (i.e., the border symmetry system) can behave as if three symmetry systems operate in a wing. These results suggest that if one removed the central symmetry system experimentally during development, the border symmetry system could compensate for the removed central symmetry system in the course of evolution of *Callicore* and *Diaethria*. This is reminiscent of the developmental plasticity of physical damage repair observed in so-called regulative eggs in contrast to mosaic eggs in experimental embryology in the 19th century [55]. The essence of this repair system is that a part can generate the whole. The *Callicore* and *Diaethria* color pattern system may be considered a natural experiment in evolution that demonstrates this plasticity. This plasticity or functional compensation is possible because of the self-similar nature of color pattern systems. The developmental plasticity of organisms in general may originate in the self-similar nature of developmental programs.

A molecular study has shown that CRISPR-Cas9 mosaic knockout of *WntA* eliminated the central symmetry system in *Junonia coenia*, *Vanessa cardui*, and *Pararge aegeria* [21], but contrary to the cases of *Callicore* and *Diaethria* above, the border symmetry system did not expand proximally. This may be because developmental plasticity is not retained in *Junonia* and *Vanessa*. In the induction model [10,13,16,17], which presumes long-range morphogenic signals in addition to molecular signals such as *WntA*, molecular signals are placed downstream of a wing-wide signaling cascade. If the induction model is correct, the result of the *WntA* knockout is not surprising.

### 4.8. The Marginal Band System and the Wing Root Band System

The wing root band is present only in certain butterflies, including *Callicore* species. The analyses of *Callicore* showed that the marginal band system and the wing root band system are continuous, suggesting that they originate as a single system. If so, because the marginal band system is a half-symmetry system (in contrast to a full-symmetry system), the wing root band system may be considered a half-symmetry system. Therefore, the wing root band may be composed of a marginal wing root band (MWB) (corresponding to MB) as the core element and a submarginal wing root band (SWB) (corresponding to SMB) as the paracore element.

An alternative interpretation is that the marginal band system may cover the outer and hind margins together, but the wing root band system may cover only the very base of the wing that is directly connected to the thorax. This structure is consistent with the illustration of the nymphalid groundplan of Otaki (2020) [12] regarding the wing root band system, but the band expression in compartment 3A is not illustrated because of its rarity. Considering that the wing root band system is directly connected with the thorax, there is a possibility that the organizer for this system may be the organizer for the entire wing system (Section 4.10). 

### 4.9. Self-Similar Configuration of the Nymphalid Groundplan

**Figure 10:**
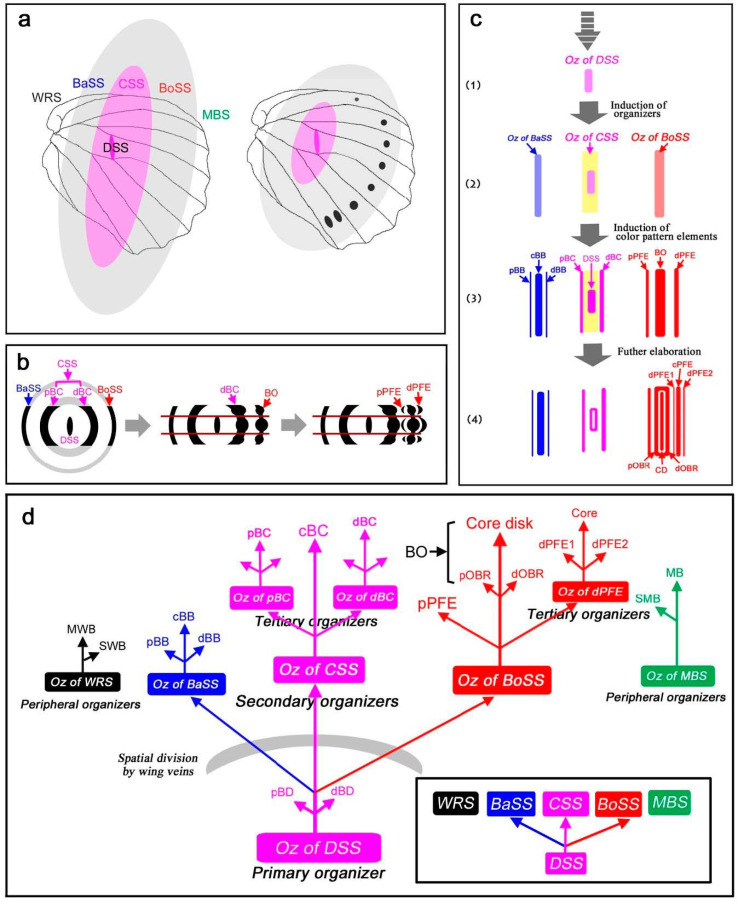
Possible relationships and developmental mechanisms of the color pattern symmetry systems. Oz: organizer, CD: core disk, and OBR: outer black ring. For other abbreviations, see Figure 1b. (**a**) Signals for wing-wide color patterns from the DSS organizer. (**b**) Developmental relationships among the three symmetry systems and parafocal elements. (**c**) Developmental order of the symmetry systems. (**d**) A tree diagram of the developmental relationships among the symmetry systems. The inset summarizes simple relationships among symmetry systems.

Based on the results of the present study, a self-similar configuration can be summarized in Figure 10 as developmental time series. This self-similarity is achieved by the repetition of the core-paracore relationship. As shown in Figure 10a, the overall configuration is determined by a putative signal from the primary organizer, which likely corresponds to the center of the discal symmetry system. The location of the discal symmetry system is determined largely by the wing veins. This finding supports the view that the discal spot is determined first, with the highest hierarchical status (aside from the potential status of the wing root band system; see Section 4.10). Signals are released from this organizer, forming multiple elongated ovals. Thus, all the symmetry systems can be understood as a single unified system (the supersymmetry system), similar to a large single eyespot with a pair of parafocal elements. The signals specify the locations of the border and basal symmetry systems, but they originate as distal or proximal components of the same signal. 

As shown in Figure 10b, the central symmetry system is composed of “an outer black ring” that is divided into distal and proximal portions (i.e., dBC and pBC, respectively). Similarly, the border and basal symmetry systems are considered distal and proximal “parafocal elements”, respectively. Figure 10b also shows how the border symmetry system expands by itself after the original signal is settled. The border symmetry system itself forms an enlarged core element and a pair of parafocal elements. These sequential steps involve the serial induction of organizers (Figure 10c,d). A tree diagram of serial induction (Figure 10d) illustrates that there are three levels of organizers. The tertiary organizers for the elaboration of dPFE, dBC, and pBC are seen in a limited number of butterflies. In this diagram, a single organizer induces three (or two) subsequent organizers: one organizer for a core element and a pair of organizers for a pair of paracore elements. The order and directions of induction may explain not only the core-paracore rule and the generalized self-similarity rule but also the DS protection rule (a paracore signal cannot be positioned directly on a core signal), the DS-CSS positioning rule (an application of the core-paracore rule), the generalized inside-dark rule (the signal is stronger inside), the field coloration rule, and the distal elaboration rule (the proximal paracore signal interferes with the original signal due to its propagation in opposing directions; see Section 4.10).

### 4.10. The Status and Possible Mechanisms of the Distal Elaboration Rule

In contrast to dPFEs, pPFEs with a bluish structural color are rare. Thus, the border symmetry system is structurally asymmetric in most cases. This rule (i.e., the distal elaboration rule) is also likely applicable to other symmetry systems, and it is somewhat contradictory to the color symmetry rule, according to which a given system is symmetric in coloration (hence referred to as the symmetry system) [10]. However, both can be considered correct. The color symmetry rule is a rough rule, and it is about color positioning and not about morphology of the system. That is, a given symmetry system often has symmetric color patterns but very asymmetric structures. In contrast, the distal elaboration rule may be considered a finer rule than the color symmetry rule; the distal elaboration rule is more about structural asymmetry.

**Figure 11:**
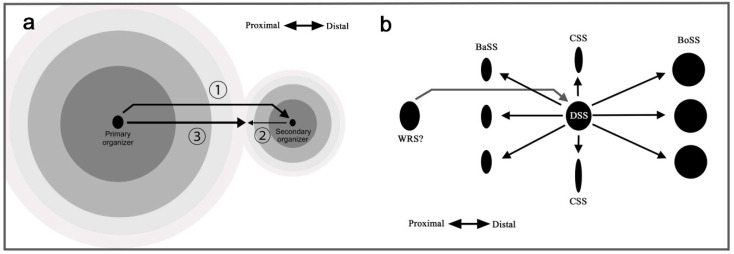
Possible mechanism that results in the distal elaboration rule. (**a**) Signal propagation from the primary organizer induces the secondary organizer (1). The secondary organizer produces the signal into both the distal and proximal sides (2). Only the signal that is propagating proximally collides with the signal from the primary organizer (3). This results in symmetry breaking of system’s structure, but not its relative color positioning. (**b**) Possible developmental relationships among system organizers. To realize the distal elaboration rule in the wing-wide supersymmetry system (i.e., BoSS is more elaborated than the BaSS), an upstream organizer should be located proximally, which may be located in or close to the thorax. The wing root band system may be qualified for this function. For abbreviations, see Figure 1b.

Because the distal portion of a wing is always wider than the proximal portion due to the fan shape of the wing, this rule may solely reflect the shape of the wing. However, considering that there are exceptions to this rule (Figure 9), one of which (Figure 9b; and maybe many others) might have evolved as satyric mimicry [56], the origin of this rule may not be so simple. Instead, this rule may be an outcome of a developmental mechanism; if the serial induction process of color pattern determination is self-similar, a signal for the proximal paracore element (i.e., pPFE) from an organizer (i.e., BO organizer) may be antagonized by a signal from the upper-level organizer (i.e., CSS organizer) because these two signals have to propagate in opposing directions (Figure 11a). This could be a mechanism of how elemental configuration (which is supposed to be originally symmetric) is modified to be asymmetric. In other words, symmetry breaking of the butterfly symmetry system may be rooted in the spatial relationship between the primary and secondary organizers.

According to this mechanism, the distal elaboration of paracore elements results from a colliding or repulsive nature of signals. For confirmation, signals often fuse together, but they have been proposed to repulse each other under different conditions [51,52]. The DS area protection rule is likely to occur similarly because the DS area is occupied by the signal from the DSS organizer earlier than the signal from the BoSS organizer even if the DSS is not expressed. When one signal arrives at a certain area, the signal can occupy that area and become dominant there. Another signal that arrives at the same area cannot settle there. More generally, a paracore signal cannot be placed on an area occupied by a core signal. This dynamic nature has been demonstrated in physical damage experiments; when the adjacent large eyespot was reduced in size by physical damage, the small eyespot became larger [51]. One could argue that the timing of the signal release may be important. Along this line of discussion, exceptional cases of the distal elaboration rule (Figure 9) are possible if the primary organizer quickly deteriorates after the specification of the secondary organizer, and the secondary organizer is active long after the cease of the signal from the primary organizer. 

To produce an asymmetric pair of PFE from the secondary organizer (in this case, BO organizer), the primary organizer is the DSS organizer (Figure 10). To produce an asymmetric pair of the basal and border symmetry systems from the secondary organizer (in this case, the DSS organizer), the primary organizer should be located proximally to the discal symmetry system. It can be speculated that the primary organizer is located at the wing base; a candidate is the organizer for the wing root band system (WRS) (Figure 11b). In this scheme, the signal from the WRS organizer specifies the location of the DSS organizer, which then specifies the CSS, BaSS, and BoSS organizers as the supersymmetry system. 

### 4.11. The Fractal Geometry of the Nymphalid Groundplan

Based on the generalization of the self-similarity rule to the wing-wide color patterns, the butterfly wing color patterns may be considered a biological fractal. Because the signal itself specifies a source, as proposed in the induction model for positional information [13,16,17], the fractal pattern may be repeated a number of times during development, at least theoretically. According to the central maxima rule, the center of an element contains the largest scales (and, thus, likely the largest cells) [31,32,57]. Similarly, pupal cuticle spots exist only at the top of the organizer [58,59]. Since organizers are bulges and dents in pupal wing tissues, butterfly wings are indeed three-dimensional [58,59,60]. The triadic Koch curve, which is a well-known fractal curve [24], is coincidentally reminiscent of the three dimensionality of butterfly wings. The Cantor ternary set (the Cantor bar) [26] is also interesting in the context of butterfly color patterns.

Self-reproduction is an essential characteristic of biological entities. At the cellular level, self-reproduction corresponds to simple unequal cell division or budding. For multicellular organisms, this corresponds to the reproduction of offspring. To reproduce, a self-similar program must be repeated. To generate complex organisms, a repeated self-similar recall program may be executed, and it may be modified based on the number of iterations. When self-similar programs are executed locally in slightly different locations in parallel, similar structures will emerge (often referred to as modularity). A self-similar program probably involves multiple levels of iterations instead of the simple co-optional use of an evolutionarily old network. It is likely that butterfly wings take advantage of self-similar recall programs to produce a complex esthetic fractal geometry.

Although the mechanisms producing the biological fractal in butterflies are not known, recent physiological experiments have revealed that the morphogenic signal is long-range and dynamic; two signals merge or interfere with one another [51,52]. Synergistic interactions appear to occur [61]. Live organizers have been observed in vivo [60]. Interestingly, signal propagation likely requires extracellular matrix as a mechanical support [62,63]. This line of physiological studies on morphogenic signals may eventually identify the long-range signals proposed in this study and reveal a link to the fractal nature of the signals. Additionally, since serial induction proceeds according to time, developmental time series events should be clarified. It should also be possible theoretically that there is a gene that is responsible for all symmetry system. Knockout butterflies of this gene may lack all three symmetry systems if not lethal.

## 5. Conclusions

In this study, a self-similar configuration of the nymphalid groundplan was revealed. The entire pattern is hierarchically constructed, forming a unified supersymmetry system. Some color pattern rules are newly proposed or generalized. Importantly, they are mostly understood based on self-similar induction processes during development. Asymmetric morphology of a symmetry system may originate from symmetry breaking during the induction processes. The precise mechanisms responsible for the construction of self-similarity are not known, but the self-similar configuration suggests that organizers are serially induced during development from primary to tertiary. The nymphalid groundplan is thus considered to be a biological fractal. The distortion hypothesis and its associated induction model [10,13,16,17] may be able to conceptually explain how this fractal configuration is produced through a serial induction process.

## Data Availability

Data sharing is not applicable to this article.
